# Year 2022: exploring COVID-19 pandemic in children

**DOI:** 10.1186/s13052-023-01536-2

**Published:** 2023-09-29

**Authors:** Elena Bozzola, Carlo Caffarelli, Francesca Santamaria, Giovanni Corsello

**Affiliations:** 1 Pediatric Unit, Bambino Gesù Children’s Hospital, IRCCS, Rome, Italy; 2 Department of Medicine and Surgery, Clinica Pediatrica, Azienda Ospedaliera-Universitaria, University of Parma, Parma, Italy; 3 Department of Translational Medical Sciences, Federico II University, Naples, Italy; 4 Department of Sciences for Health Promotion and Mother and Child Care “G. D’Alessandro”, University of Palermo, Palermo, Italy

**Keywords:** SARS-CoV-2, COVID-19, Children, Prevention, Diagnosis, Transmission, Treatment, Vaccination, Health care

## Abstract

COVID-19 pandemics is rapidly changing. In this article, we review progresses published in the Italian Journal of Pediatrics in 2022. More data on clinical pictures, prevention strategies and active management in children have been provided. The continued evolution of knowledge has driven transformations in the clinical approach to the disease and allowed key advancements in the care of children with COVID-19.

## Introduction

Since its first description in December 2019, COVID-19 pandemic continued to spread across the globe, increasing our knowledge on Sars-Cov2 and on its consequence on human health. In particular, the initial impression that minors might be rarely infected by the virus was replaced by a more nuanced understanding of infectious manifestations in children across Countries and by the recognition of disease presentation.

Aim of the review is to explore the state of knowledge on COVID-19 in children on the base of published literature in the Italian Journal of Pediatrics during 2022 year.

## Material and methods

For the purpose of the study, reports published in Italian Journal of Pediatrics from January 2022 till December 2022 and concerning COVID 19 pandemics have been examined. Identified key words to perform the research process were: SARS-CoV-2; COVID-19; children; prevention; diagnosis; transmission; treatment; health care facilities; behaviour.

Researchers focused their studies on understanding clinical findings as well as in discussing influence on other diseases, preventive strategies and therapeutical approach.

## Results

According to the search strategy, 29 articles have been included in the revision.

Evidence confirmed that in children the acute clinical presentation is generally milder than in adults. Children infected by SARS-CoV-2 even when symptomatic generally clear the virus and recover within a few days [1]. Symptoms may vary depending on age and on virus’ variants, generally including fever, rhinorrea, cough, vomiting, diarrhoea and sore throat. Several cutaneous manifestations related to COVID-19 infection have been already observed, including perniosis-like or chilblain-like lesions, characterized by vascular lesions due to microthrombosis and endothelial inflammation [2–4]. Unusual presentation have been described in case reports, including paralysis of the facial nerve in the acute phase or a few weeks after as well as abducens nerve palsy [5–7]. As for laboratory findings, most children with COVID-19 has a normal white blood count; lymphopenia is rare while neutropenia seems to be one of the altered parameters recorded more in paediatric cases than in adults [8–10].

Even if children were asymptomatic or affected by mild symptoms, they may as well experience long term sequelae as well as adult [11–13]. In details, two sequelae of paediatric COVID-19 have been identified: the multisystem inflammatory syndrome in children (MIS-C) and the long COVID [13]. MIS-C is a rare but severe hyperinflammatory disease that affects paediatric patients, typically 3–6 weeks after SARS-CoV2 contact. Most patients are previously healthy children, without relevant comorbidities [14] cardiovascular system represents the most common target organ in patients with MIS-C with electrocardiographic abnormalities and increased cardiac laboratory indices, including troponin T and NT, pro BNP levels and the risk of compromised heart function [15–17]. The cardiac involvement may be so severe to require intensive care unit, long hospitalization, and an aggressive therapy [18].

The Italian Intersociety Consensus recommends to look for symptoms suggestive of long COVID near the end of the acute phase of the disease as well as 4–12 weeks from this [13]. Long-Covid 19 may develop regardless the acute disease course and the gender even if being symptomatic during the SARS-CoV-2 infection increased six folds the risk of having at least one symptom of long COVID-19, [12, 19, 20]. Evidence suggests that long COVID have specific clinical characteristics [21, 22]. Symptoms suggestive for Long COVID in children and adolescents include persistent headache and fatigue, sleep disturbance, difficulty in concentrating, abdominal pain, myalgia or arthralgia. According to the Consensus, possible symptoms of long COVID include persistent chest pain, stomach pain, diarrhoea, heart palpitations, and skin lesions [13]. Older age and high body max index correlate to an increased risk for persistent symptoms after SARS-CoV-2 infection in children [11, 20, 23]. Children 0–5 years old had a higher risk of respiratory symptoms, while adolescents of neuro-psychological Long COVID-19 symptoms [12].

Apart from physical impairment, mental problems should be kept in mind. Bloise et al. reported that almost half of infected children presented with psychological symptoms and one out of five long-term sequelae [11]. As Gatta and al noted, there was a great increase in eating disorders, suicidal and self-harming attempts [24].

According to these findings, after COVID 19 onset, despite a great decline of paediatric emergency department admissions, there was a significant increase in patient access for neuropsychiatric diseases, in particular suicidal ideation, depression, eating disorder and psychosis. An increase in hospitalization was observed as well, mainly referring suicidal ideation, depression, eating disorder and drug abuse [25–27]. Moreover, throughout the period of infection, children spent most of the time on devices like tv-video, social media and mobile phone for non-educational activities [11]. Evidence suggest that minors look for pro-Ana materials on TikTok, Twitter or on other social media platforms [28]. Images or videos may also lead users to emulate related behaviours with an increased risk of eating disorders, suicidal or self-injury [29–31]. So, it is important to investigate the impact of the infection on children’s mental health even in absence of physical impairment and to promote nutritional and psychological support [32, 33].

With regard to self-injuring, suicidal behaviour more likely occurred during the second wave of the COVID-19 pandemic [26, 34]. In line, pharmacological therapies prescription increased to more than 80% of inpatients during the COVID year, mainly neuroleptics [24].

### Influence on other diseases

Of note, COVID-19 pandemic had also an indirect effect on other diseases, interfering with their epidemiology and diagnostic approach. For example, a strong impact on the diagnosis and management of diabetes in children was noted, revealing a more severe onset presentation compared to pre-pandemic period [35–37]. A significant reduction of stimulating tests investigating growth hormone secretion in short children have been described in a paediatric endocrinology outpatient department. Although these children might have undergone the exam and eventually treated at a later stage, it is known that the efficacy of treatment is higher when the diagnosis is prompt. In case of a delay in the diagnosis and therapy, the risk of an adult short stature is high [38]. Opposite, evidence suggests that there was an increment of precocious puberty during the pandemic period compared to previous period [38–42]. Additionally, central precocious puberty had been found linked to sleep disturbances even if the relationship has been not fully explained [39]. As evidenced by Mutlu et al., girls referred for early puberty during COVID-19 pandemic were younger than in the previous period. Of note real precocious puberty was more prevalent than pubertal variants, so that the need for pubertal suppression therapy had increased as expected [43].

Again, Mameli et al. demonstrated that COVID-19 pandemic altered the epidemiology of acute respiratory tract infections in children aged 0–5 years, with toddlers younger than 12 months of age at a higher risk [44]. Hygiene measures, social distancing, and masks by the older children may have influenced the results as well [45, 46]. Finally, Covid-19 pandemic had also an impact on reproductive health, perinatal period, and childbirth. In Iran, the incidence of congenital birth anomalies had been found significantly increased during pandemic period, mainly referring to congenital anomalies of the central nervous and genitourinary systems [47–49]. Many factors might have contribute to congenital abnormalities, including chronic maternal stress, decreased routine access to prenatal care for foetal screening and diagnosis and poverty [47].

### Prevention and therapy

Children with some specific chronic diseases are considered at risk of a severe disease course. The Italian Society of Pediatrics together with affiliated Scientific Societies recently proposed a Consensus document to detail which patients affected by comorbidities may be benefit for monoclonal antibodies administration [50–52]. As for the others, it is not possible to identify which ones may develop severe clinical manifestations, long COVID or psychosocial problems [21, 53]. Allergic rhino conjunctivitis and asthma, if under control, do not represent risk factors for the susceptibility to SARS CoV2. (54i) Patients with allergies do not present a more severe course than those without in line with the hypothesis of a positive role exerted by the Th2 immune response in COVID-19 pathogenesis [54–56].

Researchers agree on the key role of vaccination to prevent Sars Cov2 infection and, most of all, severe disease. According to The manifesto of Pediatricians of Emilia-Romagna region vaccines against COVID are the most effective and safe tool to contrast COVID 19 and for this reason should be considered as a right of children to protect themselves [57]. Vaccine product-related reactions are seldom reported such as Henoch-Schoenlein purpura reported in an adolescent by Casini et al. [58]. Nonetheless, the benefits of vaccination greatly outweigh the risks [57, 59]. Moreover, the likelihood of severe reactions such as anaphylaxis to COVID-19 mRNA vaccines in children seems very low [60, 61]. In case of previous systemic reaction to COVID-19 vaccines or excipients, Scientific Societies suggest to limit contraindications to vaccination and help to safely immunize [62].

The vaccination availability together with the application of safety protocols gives the opportunity not only to prevent infection spread but also to schedule clinical controls as well as diagnostic procedures which may have been postponed in non-urgent cases, such as pulmonary function tests [63, 64].

Nevertheless, vaccination coverage in the pediatric age is actually lower compared to adults, leading minors to a higher susceptibility to SARS-CoV-2 [65]. Of notes, many parents have a positive attitude towards their children’s vaccination [66]. Factors that may influence children’s willingness to be vaccinated against COVID-19 include gender, age, residence, number of children in the household, parent education [67, 68]. Adolescents as well have been described in favour of immunisation by Cupertino et al. Parents' opinion strongly affects the immunization status of adolescents as in Cupertino et al. survey the most important predictors of being immunized against SARS-CoV2 were having both parents immunized. Of note, minors reported their willing on getting COVID 19 vaccine information by family doctors and at school, underling the potential role of paediatricians and school educators [69]. Increased information and sensibilization on the safety and efficacy of vaccination is required to increase vaccine coverage and should be supported by effective persuasion campaign for those reluctant to accept the vaccine [70].

Strength of our manuscript is to summarize the latest evidence on COVID 19 as published in the Italian Journal of Pediatrics, an open access peer-reviewed journal that includes all aspects of pediatric medicine. Limiting the search strategy to articles published in the Italian Journal of Pediatrics in 2022 represents the weakness of the present manuscript.

## Conclusions

New insights in COVID-19 continue to be presented. This is reflected by many studies on COVID-19 that have been published by Italian Journal of Pediatrics during the past year. We expect that in the coming years ongoing trials will provide options for improving prevention and for allowing an effective treatment.

## Data Availability

Data sharing is not applicable to this article as no datasets were generated or analysed during the current study.

## Abbreviations

COVID-19: Coronavirus disease of 2019
SARS-CoV-2: Severe acute respiratory syndrome coronavirus
MIS-C: Multisystem infammatory syndrome in children
